# Complete Genome Sequence of an Extensively Drug-Resistant *Shewanella xiamenensis* Strain Isolated from Algerian Hospital Effluents

**DOI:** 10.1128/genomeA.01236-16

**Published:** 2016-11-10

**Authors:** Khadidja Yousfi, Abdelaziz Touati, Sadjia Bekal

**Affiliations:** a Laboratoire d’Écologie Microbienne, FSNV, Université A/MIRA de Bejaïa, Algeria; b Laboratoire de Santé Publique du Québec, Institut National de Santé Publique du Québec, Sainte-Anne-de-Bellevue, Québec, Canada; c Département de Microbiologie, Infectiologie et Immunologie, Université de Montréal, Montréal, Québec, Canada

## Abstract

In this study, we present the first complete genome of an extensively drug-resistant strain of *Shewanella xiamenensis*, collected from hospital effluents in Algeria. This genome includes the chromosome and a large new plasmid harboring several drug-resistance genes.

## GENOME ANNOUNCEMENT

*Shewanella xiamenensis* is a recently validated species (1) that has been reported in different parts of the world (2–6). Bacteria of the genus *Shewanella* are motile Gram-negative bacilli belonging to the *Shewanellaceae* family. They have been proposed to be a reservoir and vehicle of different antibiotic-resistance determinants (2, 3, 6–8). In *S. xiamenensis*, the *bla*_OXA-48_-like genes encoding carbapenem-hydrolyzing enzymes have been reported and the bacterium is recognized as the progenitor of these genes (2–5). Recently, different resistance genes and mobile genetic elements have been identified in *S. xiamenensis* (6, 8).

Here, we present the first complete genome of *S. xiamenensis* T17, including the chromosome and a multidrug-resistance plasmid (pSx1). This extensively drug-resistant (XDR) isolate produces extended-spectrum β-lactamase and is resistant to penicillin, ampicillin, piperacillin, ticarcillin, cefotaxime, ceftazidime, cefepime, ertapenem, aztreonam, gentamicin, streptomycin, ciprofloxacin, trimethoprim-sulfamethoxazole, tetracycline, azithromycin, and chloramphenicol.

This XDR strain of *S. xiamenensis* was isolated from hospital effluents in 2012 in Tizi Ouzou, Algeria. The whole-genome sequencing was performed using the PacBio SMRTbell template prep kit version 1.0 (Pacific Biosciences, Menlo Park, CA, USA). The assembly of the long reads was done using the Hierarchical Genome Assembly Process (HGAP) version 3, and the high-quality unitigs were generated with the Quiver algorithm. The sequence annotation was performed by Prokka version 1.11. To our knowledge, only one draft genome of *S. xiamenensis* (strain BC01) is deposited in GenBank (accession number JGVI00000000) (9).

The chromosome size of *S. xiamenensis* T17 was 5,084,350 bp, and the plasmid size was 268,412 bp. A total of 4,587 open reading frames were detected on the chromosome and 297 on the plasmid. The *bla*_OXA-416_ gene—a variant of the recently described *bla*_OXA-48_ gene, which confers resistance to carbapenem—was detected in the chromosome of T17 (5). To date, plasmids identified in the genus *Shewanella* do not carry any drug-resistance genes. Analysis of this new plasmid showed the presence of *bla*_VEB-16_ and *bla*_PSE-1_ conferring resistance to β-lactams, *qnrVC6* to quinolones, *dfrA12* and *dfrA23* to trimethoprim; *aacC1*, *aac(6)-II*, *aadA1*, and *aadA2* to aminoglycosides; *tetR*, *tetA*(C), and *tetA*(G) to tetracyclines; *sul1* and *sul1Δ* to sulfonamides; *catA2* and *cmlA9* to chloramphenicol; *msr(E)* and *mph(E)* to macrolides; and *qacG* to quaternary ammonium compounds. The *acrA*, *acrB*, and *oep* multidrug efflux pumps were also detected. These genes are carried by a new Tn*1696* transposon derivative associated with several mobiles genetic elements. The mercury-resistance system, including *merE*, *merD*, *merB*, *merA*, *merP*, *merT*, and *merR*, was also identified (6). This high-quality genome sequence could be used as a reference for assembly genomes and could be investigated in different studies on *Shewanella* spp. and the evolution and transfer of resistance genes.

### Accession number(s).

This whole-genome shotgun project has been deposited at DDBJ/EMBL/GenBank under the accession number LDOA00000000. The version described in this paper is the first version, LDOA01000000.
